# Retinal vasoproliferative tumor in intermediate uveitis: A case report

**DOI:** 10.1016/j.amsu.2020.08.039

**Published:** 2020-09-06

**Authors:** Ahmed Mahjoub, Feriel Ammar, Nadia Ben Abdesslam, Tasnim Mhamdi, Leila Knani, Mohamed Ghorbel, Hachmi Mahjoub

**Affiliations:** Department of Ophtalmology, Farhat Hached Hospital, Sousse, Tunisia

**Keywords:** Vasoproliferative tumor –intermediate uveitis, Ocular inflammation

## Abstract

**Introduction:**

Retinal vasoproliférative tumors (VPTs) have been reported as uncommon complications of intermediate Uveitis.

**Case description:**

A patient consulted for a gradually decreased vision in the right eye (RE). The examination of the RE found a corrected visual acuity at 20/100 with normal eye pressure measured. The anterior segment was deep and quiet and 2+ vitreous haze was found. Funduscopy showed a VPT. Retinal fluorescein angiography of the RE revealed macular cystoid edema also objectified by the Optical Coherence Tomography (OCT). Intermediate Uveitis was considered idiopathic. The patient received a cryoapplication and was put on oral corticosteroid therapy with improvement of visual acuity and a regress of both vitreous inflammation and cystoid macular edema.

**Conclusion:**

The association of a VPT with intermediate uveitis represents a real diagnostic and therapeutic challenge and imposes rigorous care and monitoring strategy combining internist and ophthalmologist.

## Highlights

• Retinal vasoproliferative tumors (VPTs) have been reported as uncommon complications of intermediate Uveitis.
• The presence of active intermediate uveitis accompanied by VPTs suggests the need for an aggressive uveitis treatment.
• The causative process underlying the development of VPT in eyes with intermediate Uveitis remains unknown.
• We speculate in this case report a pathogenic relationship of this long-standing inflammation to the vascular tumor.

## 1 Introduction

Retinal vasoproliferative tumors (VPT) have been described for the first time in 1983 under the name of “Presumed Acquired Retinal Hemangiomas” [1]. They are rare and benign tumors, which can be idiopathic or secondary to other eye pathologies [2].The clinical diagnosis is based on the visualization of a yellowish lesion with indistinct edges at Funduscopy, associated with telangiectasia of the peripheral retina and intraretinal exudates [3].We report here a case of (VPT) complicating intermediate uveitis. This case has been reported in line with the SCARE criteria [4].

## 2 Case report

A 37-year-old patient, with no prior medical history, consulted for a gradually decreased vision in the right eye (RE) evolving for a month, without other associated signs.

The examination of the RE found a corrected visual acuity at 20/100 with normal eye pressure measured at 12 mmHg. The anterior segment was deep and quiet and 2+ vitreous cells was found.

**Fig. 1:**
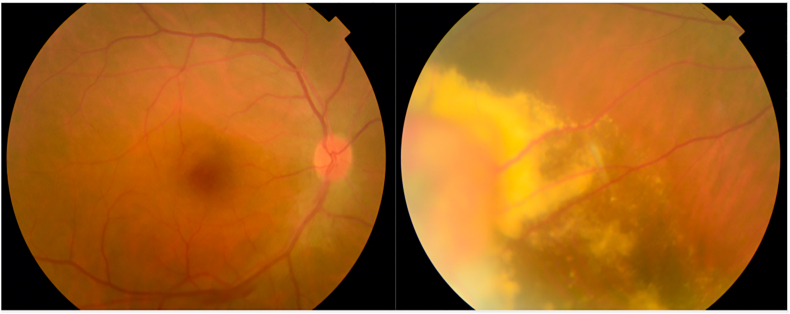
Funduscopy of the RE showing multiple exudates of the peripheral retina surrounding a raised yellow-orange lesion. (For interpretation of the references to colour in this figure legend, the reader is referred to the Web version of this article.)

Funduscopy showed multiple exudates of the peripheral retina surrounding a raised yellow-orange lesion, with blurred edges, associated with telangiectasia suggesting a (VPT) [Fig. 1].

The examination of the left eye (LE) was normal and the visual acuity was 20/20.

**Fig. 2:**
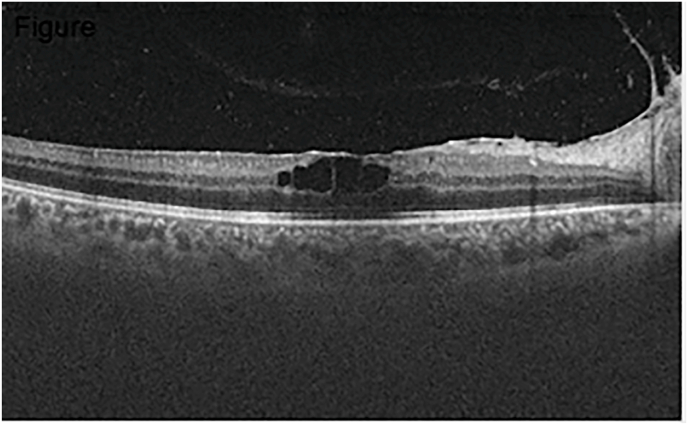
Macular OCT showing cystoid macular edema of the RE.

Retinal fluorescein angiography of the RE revealed staining in the macular area caused by macular Cystoid edema also objectified by the Optical Coherence Tomography (OCT) [Fig. 2].

**Figure:**
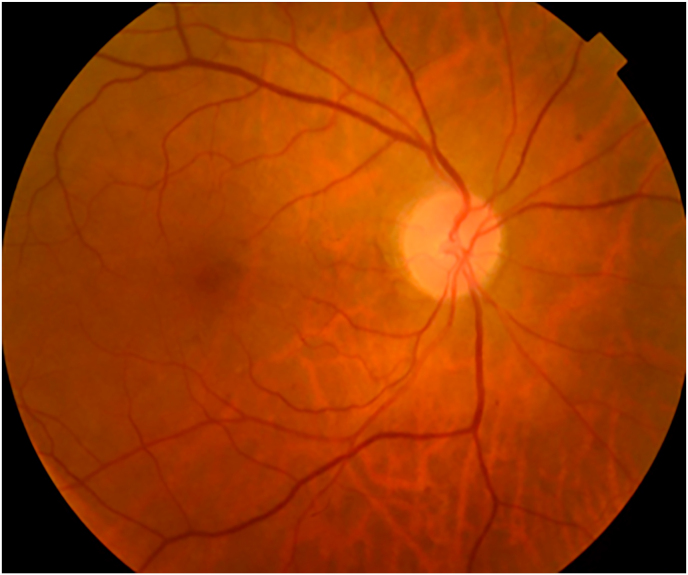

**Figure:**
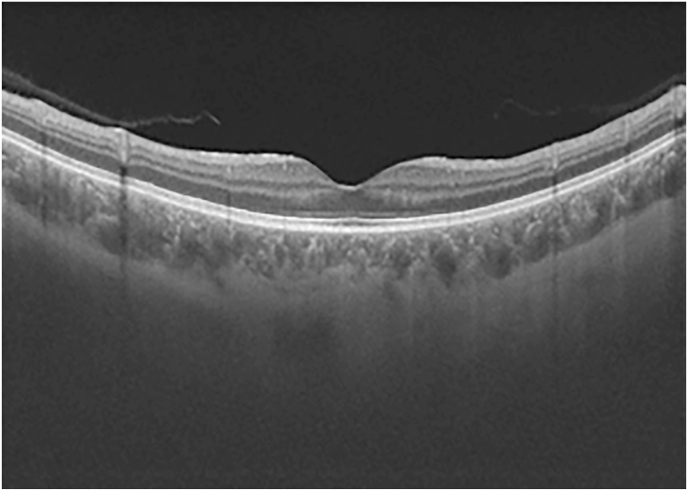

An etiological assessment of intermediate uveitis (intradermal reaction to tuberculin, serology of syphilis and toxoplasmosis, a phosphocalcic balance, an assay of the rate of serum converting enzyme, etc.) was requested but returned negative. Intermediate Uveitis was considered idiopathic.

The patient received a cryoapplication applied in a transconjunctival way, under observation through binocular indirect ophthalmoscopy. The treatment goal is to freeze tumor, allowing slow thawing and repeating the whole process 2 times. He was put on oral corticosteroid therapy at 10mg/kg/day (700 mg/day) with progressive degression throughout 45 days.

A month later, the visual acuity of the right eye improved to 20/25. The vitreous inflammation was controlled and the cystoid macular edema regressed as well as the tumor.

## 3 Discussion

Intermediate uveitis is an idiopathic and insidious syndrome which involves the pars plana, peripheral retina, underlying choroid, and anterior vitreous.It usually affects children and young adults. The clinical picture consists of vitritis with a relatively quiet anterior segment, pars plana inflammatory debris termed “snow-banking,” retinal periphlebitis, cystoid macular edema, and optic nerve edema [1].

Retinal vasoproliferative tumor is a rare disease that has capillary hemangioma as the most frequent differential diagnosis. The tumor is considered to be of reactive nature. It can be idiophatic or secondary to other ocular diseases such as: uveitis, retinitis pigmentosa, sickle cell disease, previous surgery and retinopathy of prematurity [2,3]. Retinal vasoproliférative tumors (VPTs) have been reported as uncommon complications of intermediate Uveitis.

The diagnosis of VPT is mainly clinical based on fundus examination that reveals a fibrous solid tumor located most often at the inferotemporal peripheral retina, associated with telangiectasia, retinal hemorrhages, and intra-retinal exudates. It can be associated with cystoid macular edema or serous retinal detachment [5]. When viewed, the nourishing retinal artery is significantly less dilated and less tortuous in capillary hemangiomas which is the main differential diagnosis of VPT.

Histologically, VPT has a vascular component and a glial component. Recent studies have demonstrated the predominance of the astrocytic and glial component, hence the proposal for the term “Reactive Retinal Astrocytic Tumors” [6].

VPT can be primary or secondary, mostly complicating retinitis pigmentosa and uveitis [7]. Being rare, they are less described in the literature than other complications of intermediate uveitis (macular edema, cataract, epiretinal membrane, glaucoma, etc.). Chronic inflammation of the vitreous and peripheral retinal ischemia by a sheathing of retinal veins triggers a mechanism of angiogenesis which may trigger neovascularization of the base of the vitreous, or even the formation of VPT [8]. The existence of macular edema during an episode of intermediate uveitis, although it is a very common complication, should encourage careful examination of the peripheral retina for neovascularization or possible VPT.

Despite their peripheral location, VPT can worsen the decreased visual acuity during intermediate uveitis through the caused complications, the most frequent being exudative retinal detachment and neovascular glaucoma [8]. The association of a VPT with intermediate uveitis then justifies an aggressive treatment [1].

It is important to recognize the VPT as clinically distinct lesions, which should be monitored periodically as they can lead to vision loss due to their exudative tendencies, as described in our case, and other associated complications such as vitreous hemorrhage, formation of epiretinal membrane, subretinal proliferation, macular edema, rubeotic glaucoma, etc. These lesions must be differentiated from other vascular or tumor lesions of the ocular fundus because their prognosis and treatment are different.

Several treatments are currently proposed for VPT [9]: Laser photocoagulation, cryoapplication, dynamic phototherapy, vitrectomy associated with the endo-ocular laser, etc. Intravitreal injections of antiangiogenic agents are proposed as adjuvant therapy [10]. Laser photocoagulation allows selective treatment of vascular anomalies, some authors defend its superiority to cryoapplication. Few cases of VPT treated by photocoagulation alone have been reported. An adjuvant cryoapplication is often necessary. The cryoapplication only can, however, be proposed.

## 4 Conclusion

Retinal VPT are rare. They can be idiopathic or secondary. The diagnosis is clinical, based on the evocative aspect of fundus examination. Their pathophysiology remains poorly understood. They can cause blindness by their serious complications. Several therapeutic alternatives are currently proposed. The association of a VPT with intermediate uveitis represents a real diagnostic and therapeutic challenge and imposes rigorous care and monitoring strategy combining internist and ophthalmologist.

## Provenance and peer review

Not commissioned, externally peer reviewed.

## Grant support or financial relationship

There is no funding for this study.

## Patient consent

Written informed consent was obtained from the patient for publication of this case report and accompanying images. A copy of the written consent is available for review by the Editor-in-Chief of this journal on request.

## Ethical approval

The study was approved by Ethics Committee.

## Sources of funding

This study has not received any funding.

## Author contribution

Study concept or design – AM, FA.

Data collection – AM, FA,NBA, TM.

Data interpretation – AM, FA,MG.

Literature review – FA,TM.

Drafting of the paper – FA, MG, AM.

Editing of the paper – FA, NBA,LK.

## Trial registry number

Name of the registry:
Unique Identifying number or registration ID:
Hyperlink to the registration (must be publicly accessible):

## Article guarantor

Feriel Ammar.

## Consent

Written informed consent was obtained from the patient.

## CRediT authorship contribution statement

**Ahmed Mahjoub:** Data curation, Writing - review & editing. **Feriel Ammar:** Data curation, Writing - review & editing. **Nadia Ben Abdesslam:** Writing - review & editing, Supervision. **Tasnim Mhamdi:** Data curation, Writing - review & editing. **Leila Knani:** Data curation, Writing - review & editing. **Mohamed Ghorbel:** Supervision. **Hachmi Mahjoub:** Supervision.

## Declaration of competing interest

The authors have no conflict of interest to declare.
